# Piezo1 channel activation in response to mechanobiological acoustic radiation force in osteoblastic cells

**DOI:** 10.1038/s41413-020-00124-y

**Published:** 2021-03-10

**Authors:** Guangdao Zhang, Xiaofei Li, Lin Wu, Yi-Xian Qin

**Affiliations:** 1 Department of Biomedical Engineering, Stony Brook University, Stony Brook, NY 11794 USA; 2 Department of Prosthodontics, School of Stomatology, China Medical University, Shenyang, China

**Keywords:** Bone, Bone quality and biomechanics

## Abstract

Mechanobiological stimuli, such as low-intensity pulsed ultrasound (LIPUS), have been shown to promote bone regeneration and fresh fracture repair, but the fundamental biophysical mechanisms involved remain elusive. Here, we propose that a mechanosensitive ion channel of Piezo1 plays a pivotal role in the noninvasive ultrasound-induced mechanical transduction pathway to trigger downstream cellular signal processes. This study aims to investigate the expression and role of Piezo1 in MC3T3-E1 cells after LIPUS treatment. Immunofluorescence analysis shows that Piezo1 was present on MC3T3-E1 cells and could be ablated by shRNA transfection. MC3T3-E1 cell migration and proliferation were significantly increased by LIPUS stimulation, and knockdown of Piezo1 restricted the increase in cell migration and proliferation. After labeling with Fluo-8, MC3T3-E1 cells exhibited fluorescence intensity traces with several high peaks compared with the baseline during LIPUS stimulation. No obvious change in the fluorescence intensity tendency was observed after LIPUS stimulation in shRNA-Piezo1 cells, which was similar to the results in the GsMTx4-treated group. The phosphorylation ratio of ERK1/2 in MC3T3-E1 cells was significantly increased (*P* < 0.01) after LIPUS stimulation. In addition, Phalloidin-iFluor-labeled F-actin filaments immediately accumulated in the perinuclear region after LIPUS stimulation, continued for 5 min, and then returned to their initial levels at 30 min. These results suggest that Piezo1 can transduce LIPUS-induced mechanical signals into intracellular calcium. The influx of Ca^2+^ serves as a second messenger to activate ERK1/2 phosphorylation and perinuclear F-actin filament polymerization, which regulate the proliferation of MC3T3-E1 cells.

## Introduction

Millions of fractures occur in the United States every year, with the average rate of nonunion fractures being roughly between 5% and 10%, which is predicted to increase over time.^1,2^ The risk of nonunion fracture is mainly related to several factors, including the severity of the injury and type of treatment. Currently, for the treatment of fracture or bone defects, several treatment modalities can be considered, either alone or in combination, for optimization of the bone healing process.^3^ In addition to typical approaches, such as fixation and bone transport, mechanobiological interventions have shown promise in promoting cellular proliferation and tissue adaptation; of these strategies, low-intensity pulsed ultrasound (LIPUS)^4^ and pulsed electromagnetic fields^5^ have been extensively utilized in the clinical setting to enhance bone regeneration and fresh fracture as noninvasive modalities of biophysical stimulation. The US Food and Drug Administration approved LIPUS for the acceleration of fresh bone fracture healing in 1994.^6^ Previous studies have comprehensively demonstrated that LIPUS can promote bone fracture healing and repair. The latest meta-analysis indicated that LIPUS treatment could be considered an optimal treatment modality for patients with fresh fractures because it can reduce the time to fracture union and improve quality of life.^4^ A systematic review also showed that LIPUS treatment could facilitate fracture healing by increasing bone formation in cases of delayed nonunion and impaired bone fractures.^7^

Although the effects of LIPUS are evident, the biophysical mechanisms have not been fully elucidated. Acoustic pressure waves with an energy of 30 milliwatts (mW·cm^−2^) generated by LIPUS stimulation could be delivered transcutaneously to the fracture site.^6^ For LIPUS to have a biological effect, the mechanical wave must be converted to biochemical signals that activate biochemical pathways in the cell. Intracellular calcium (Ca^2+^) signaling, which acts as a secondary messenger toward the activation of various cellular functions, is one of the earliest events in mechanotransduction.^8^ The sources of Ca^2+^ elevation induced by mechanical stimulation have been demonstrated to be either extracellular Ca^2+^ from the environment or Ca^2+^ stored from areas such as the endoplasmic reticulum (ER).^9,10^ The influx of extracellular Ca^2+^ is the primary source of the rapid initial calcium influx under mechanical stimulation in osteoblasts.^11,12^ Ca^2+^ enters the cytoplasm through calcium channels in the cell membrane (such as calcium-binding proteins or voltage-gated calcium channels).

Mechanosensitive Piezo ion channels, including Piezo1 and Piezo2, are evolutionarily conserved proteins that are critical for normal physiological processes in mammals.^13,14^ Piezo1 is localized at or near the plasma membrane. Ge et al. explored the structure of Piezo1 using single-particle cryoelectron microscopy and found that Piezo1 formed a trimeric propeller-shaped structure, including three blades, a central cap, and core transmembrane segments.^15,16^ In addition, its characteristically curved blades and core transmembrane segments (central cation-selective pore) as a pivot form a lever-like apparatus, and this lever-like mechanotransduction mechanism might enable Piezo1 channels to allow cation-selective translocation.^17^ In cells, Piezo1 channels can respond rapidly to diverse forms of mechanical stimulation and convert mechanical cues into biochemical signals to modulate various physiological processes.

Piezo1 is a sensor of shear stress, and endothelial cells can be regulated to determine vascular structure and function with Piezo1-dependent shear stress-evoked ionic currents and calcium influx.^18,19^ Piezo1 also plays an important role in not only mechanical stretching, triggering rapid epithelial cell division,^20^ but also mechanotransduction of the ultrasound-stimulated response in dental stem cells.^21^ We thus hypothesized that Piezo1 may be a vital mechanotransduction component expressed on the membrane of osteoblast precursor cells (MC3T3-E1) and significantly involved in the processes of transducing ultrasound-associated mechanical stimulation signals and activating the corresponding downstream signaling pathways. Therefore, we herein investigated the expression and role of Piezo1 in MC3T3-E1 cells after treatment with LIPUS.

## Results

### Piezo1 is present on MC3T3-E1 cells and can be ablated by shRNA transfection

**Fig. 1:**
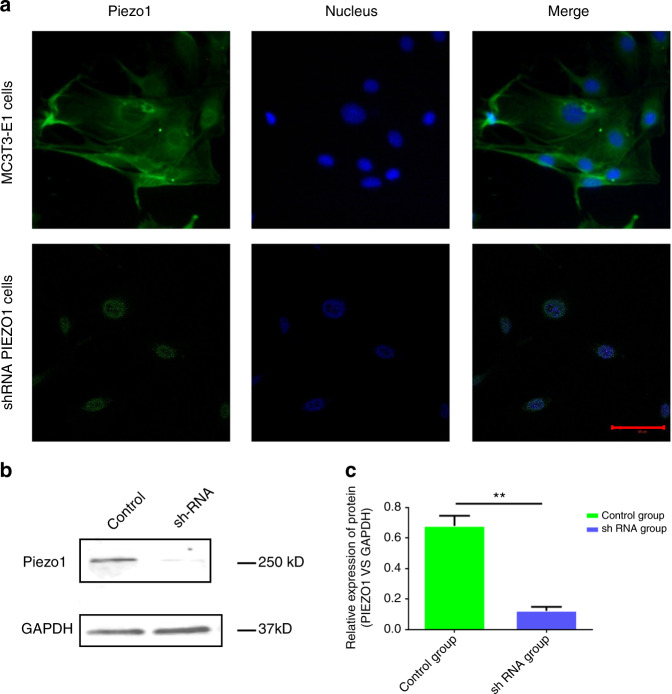
Expression of Piezo1 in MC3T3-E1 and shRNA-Piezo1 MC3T3-E1 cells. **a** Piezo1 (green) was expressed on MC3T3-E1 cells and localized at the plasma membrane and nucleus (blue). The protein expression of Piezo1 was decreased in shRNA-Piezo1 cells, especially on the plasma membrane. Piezo1 was expressed only around the nucleus in the shRNA-Piezo1 cells. The scale bar is 50 μm. **b**, **c** Western blot analysis showed that the Piezo1 protein expression in the shRNA-Piezo1 MC3T3-E1 group was significantly lower than that in the control group (*n* = 3, *P* < 0.01, Student’s *t*-test)

Immunofluorescence analyses with an anti-Piezo1 antibody were utilized to observe the expression and localization of the Piezo1 protein, revealing that Piezo1 was expressed in MC3T3-E1 cells and localized in the plasma membrane and nucleus. After Piezo1 shRNA lentiviral particle transfection, the protein expression of Piezo1 protein, especially in the plasma membrane (Fig. 1a). Western blot analysis demonstrated that the relative expression of Piezo1 [Piezo1 vs. glyceraldehyde 3-phosphate dehydrogenase (GAPDH)] was only 0.123 ± 0.025, which was significantly lower than that in the control group (0.679 ± 0.066) (*n* = 3, *P* < 0.01, Student’s *t*-test) (Fig. 1b, c, full-length western blots of Piezo1 and GAPDH expression are shown in Supplementary Fig. 1). Thus, we concluded that Piezo1 was indeed expressed on MC3T3- E1 cells, and shRNA lentiviral particle transfection knocked down its expression by more than 80%.

### MC3T3-E1 cell migration and proliferation are significantly increased by LIPUS stimulation, and knockdown of Piezo1 restricts the increase in cell migration and proliferation

The migration and proliferation of osteoblasts play a crucial role in the bone healing process. In this experiment, wound healing and migration assays were conducted to measure the rates of MC3T3-E1 and shRNA-Piezo1 cell proliferation and migration. Four hours after LIPUS stimulation, the cell-covered area of MC3T3-E1 cells was 45.91% ± 2.29%, which was significantly higher than that of shRNA-Piezo1 cells (32.82% ± 1.79%) (*n* = 3, *P* < 0.01, Student’s *t*-test). The cell-covered area continued to increase at 8 and 12 h after LIPUS stimulation in both MC3T3-E1 cells and shRNA-Piezo1 cells, and the differences were also significant at 4 h. Without LIPUS stimulation, the cell-covered area of MC3T3-E1 cells was 27.42% ± 1.42%, which was slightly higher than that of shRNA-Piezo1 cells (24.67% ± 3.43%) at 4 h, although the difference was not significant (*n* = 3, *P* > 0.05, Student’s *t*-test). At the 8- and 12-h time points, these two groups of cells showed trends similar to those at the 4-h time point.

**Fig. 2:**
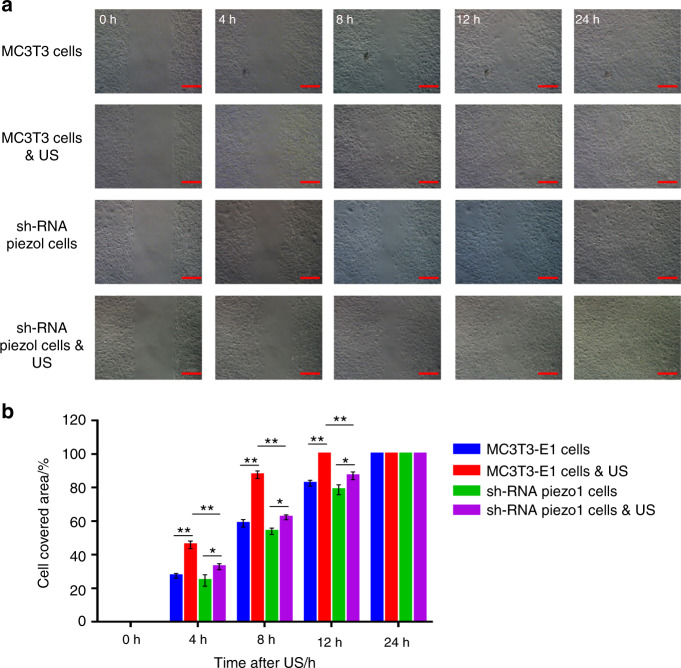
Wound healing and migration assay. **a** Randomly selected images of the gap (500 μm) at 0, 4, 8, 12, and 24 h after treatment with or without LIPUS stimulation. Scale bars, 200 μm. **b** Changes in the cell-covered area over time. LIPUS stimulation significantly increased the migration and proliferation of MC3T3-E1 cells (*n* = 3, **P < 0.01, Student’s *t*-test). LIPUS stimulation also increased the migration and proliferation of shRNA-Piezo1 cells (*n* = 3, *P < 0.05, Student’s *t*-test), but the difference was not as obvious as it was in MC3T3-E1 cells. The cell-covered area of MC3T3-E1 cells was significantly higher than that of shRNA-Piezo1 cells at 4, 8, and 12 h after LIPUS stimulation (*n* = 3, **P < 0.01, Student’s *t*-test)

In addition, there were no significant differences in the migration abilities of MC3T3-E1 cells without LIPUS stimulation and shRNA-Piezo1 cells with and without LIPUS stimulation (*n* = 3, *P* > 0.05, Student’s *t*-test) (Fig. 2a, b). Moreover, these results indicate that Piezo1 may be an important mechanosensory factor in the process of ultrasound stimulation by increasing the migration and proliferation of MC3T3-E1 cells.

### Fluorescence imaging analysis of calcium oscillation and the effects of LIPUS stimulation on different groups of cells

**Fig. 3:**
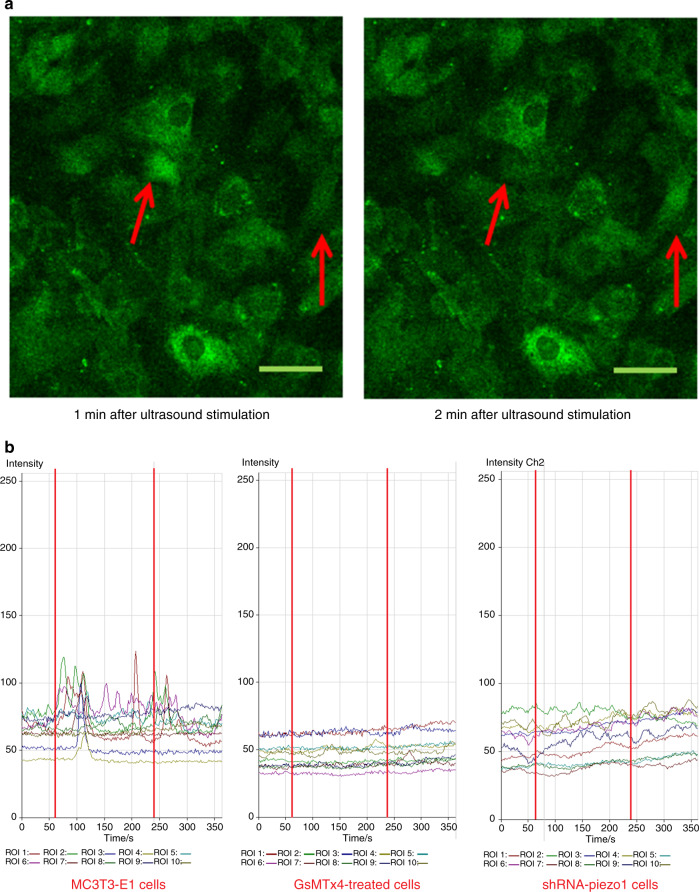
Fluorescence imaging of calcium oscillation and the effects of LIPUS stimulation on different groups of cells. **a** After LIPUS stimulation, the Fluo-8-labeled cells exhibited increased intracellular calcium levels at different time points. The red arrows show the two-cell calcium oscillation phenomenon. Scale bars, 50 μm. **b** Representative intracellular calcium traces of three groups of cells (MC3T3-E1, shRNA-Piezo1, and GsMTx4-treated cells) are shown as the fold increase in Fluo-8 intensity in response to LIPUS stimulation. The experiment was performed for six total minutes, including 1 min of baseline without LIPUS stimulation, 3 min of active stimulation (between the two red lines), and 2 min of regression. Time-lapse sequences were collected every 1.8 s for 6 min. In each field of interest, the fluorescence intensities of 10 cells were quantified using LSM Image Browser software

It is hypothesized that Piezo1 (mechanosensitive Ca^2+^ channel) in MC3T3-E1 cells might be the mechanosensor for LIPUS stimulation. Thus, Piezo1 could be activated by LIPUS stimulation and then open for intracellular calcium translocation. With LIPUS stimulation, the calcium flickers of Fluo-8-labeled cells showed oscillation of the intracellular calcium level (Fig. 3a). In all the groups in this experiment, the fluorescence intensities of ten cells were quantified. In the MC3T3-E1 cell group, the fluorescence intensity traces exhibited several high peaks compared with the baseline during LIPUS stimulation (between the two red lines). After treatment with LIPUS, the fluorescence intensity peaks were still present during the first minute of the regression period and then gradually returned to baseline at the end of the experiment (Fig. 3b, left). In the GsMTx4-treated cell group, with all the cationic mechanosensitive channels (MSCs) inhibited by GsMTx4, no obvious changes in the fluorescence intensities were observed after LIPUS stimulation, and the peak values remained almost constant from 0 to 6 min (Fig. 3b, middle). However, in the shRNA-Piezo1 cell group, some small peaks of fluorescence intensity were observed from 1 to 5 min. However, the peaks were not as high as those in the MC3T3-E1 cell group. The changes in the fluorescence intensity traces in the shRNA-Piezo1 cell group were similar to those in the GsMTx4-treated cell group (Fig. 3b, right). Thus, we concluded that Piezo1 is one of the most important Ca^2+^ channels, but not the only one, in MC3T3-E1 cells, and that it can be activated by ultrasound stimulation.

### Piezo1 can transduce LIPUS-associated mechanical signals and activate ERK1/2 phosphorylation

**Fig. 4:**
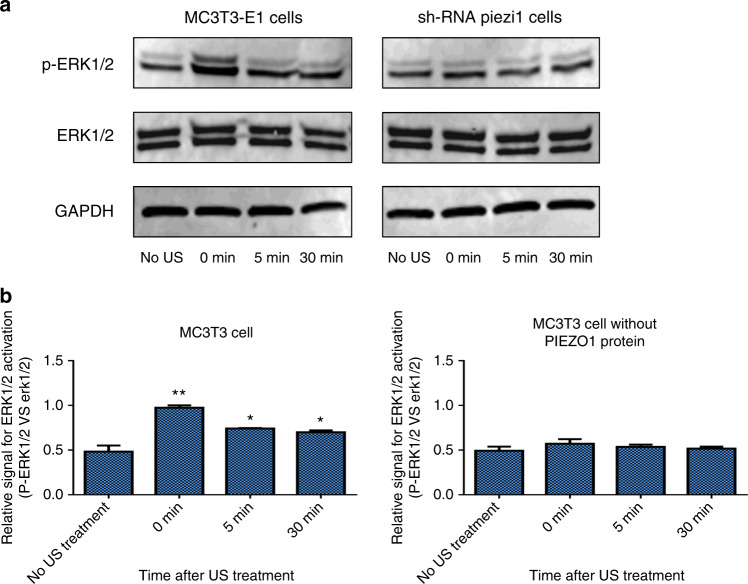
Western blot analysis of ERK1/2 and p-ERK1/2 in MC3T3-E1 and shRNA-Piezo1 cells after LIPUS stimulation. **a** Representative western blots of ERK1/2, p-ERK1/2, and GAPDH in MC3T3-E1 and shRNA-Piezo1 cells at the indicated time points (0, 5, and 30 min) after LIPUS stimulation. **b** Quantitative changes in ERK1/2 activation in MC3T3-E1 and shRNA-Piezo1 cells. The ratio of ERK1/2 phosphorylation to the relative expression of the protein doublet (p-ERK1/2 vs. ERK1/2) is presented as a parameter of ERK1/2 activation (*n* = 3, *P < 0.05, **P < 0.01, Student’s *t*-test)

ERK1/2 is a signaling molecule that is widely known to be both US-activated and calcium-mediated, and its phosphorylation is the trigger for osteoblast proliferation.^22,23^ The expression levels of ERK1/2 and p-ERK1/2 in MC3T3-E1 and shRNA-Piezo1 cells were determined via Western blot (Fig. 4a). The ratio of phosphorylated ERK1/2 to ERK1/2 (p-ERK1/2 vs. ERK1/2) in the MC3T3-E1 cell group was significantly increased from 0.483 ± 0.069 before LIPUS stimulation to 0.975 ± 0.026 after LIPUS stimulation (*n* = 3, *P* < 0.01, Student’s *t*-test).

After 5 and 30 min of LIPUS stimulation, the phosphorylated ratios of ERK1/2 were decreased to 0.742 ± 0.005 and 0.700 ± 0.021, respectively, which were still higher than that in MC3T3-E1 cells without LIPUS stimulation (*n* = 3, *P* < 0.05, Student’s *t*-test). In the shRNA-Piezo1 cell group, the phosphorylated ratio of ERK1/2 was increased only from 0.493 ± 0.046 to 0.571 ± 0.050 after LIPUS stimulation, and the difference was not significant (*n* = 3, *P* > 0.05, Student’s *t*-test) (Fig. 4b). These results indicate that the activation of ERK1/2 in MC3T3 cells is related to LIPUS stimulation and that PIEZO1 might act as the mechanosensor in this process.

### LIPUS stimulation induces the polymerization of perinuclear F-actin with the Piezo1 mechanosensor in MC3T3-E1 cells

**Fig. 5:**
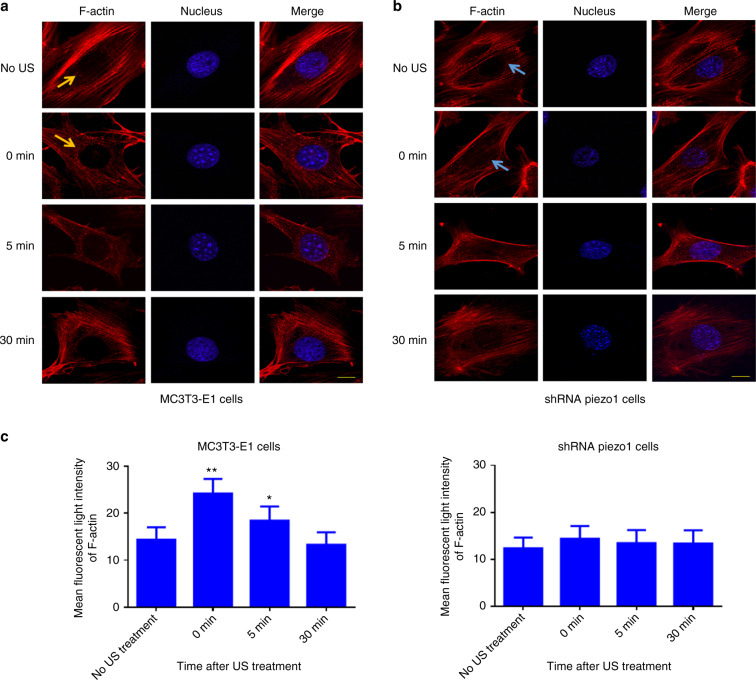
Polymerization of perinuclear F-actin after LIPUS stimulation. **a**, **b** Representative fluorescence images of Phalloidin-iFluor-labeled F-actin around the nuclei in MC3T3-E1 and shRNA-Piezo1 cells. The orange arrows in **a** and blue arrows in **b** indicate perinuclear F-actin. Scale bars: 10 μm. **c** The mean fluorescence intensity of perinuclear F-actin was measured and analyzed with ImageJ at the indicated time points (0, 5, and 30 min) after LIPUS stimulation (*n* = 9, *P < 0.05, **P < 0.01, Student’s *t*-test)

To investigate how F-actin structures respond to LIPUS stimulation, F-actin was stained, and the mean fluorescent light intensity was measured and analyzed within the perinuclear regions. After 3 min of LIPUS stimulation, the MC3T3-E1 and shRNA-Piezo1 cells were fixed and stained with Phalloidin-iFluor 555 Reagent at the following time points: 0, 5, and 30 min. In the MC3T3-E1 cell group, Phalloidin-iFluor-labeled F-actin filaments were found to immediately accumulate at the perinuclear region (red granular F-actin polymer indicated by an orange arrow) more extensively than in unstimulated cells. The polymerization of perinuclear F-actin continued for 5 min and then returned to baseline at 30 min (Fig. 5a). In the shRNA-Piezo1 cell group, perinuclear F-actin accumulation was not observed (indicated by a blue arrow) (Fig. 5b). Using ImageJ software, the mean fluorescence intensity of perinuclear F-actin was measured and analyzed. In the MC3T3-E1 cell group, the perinuclear F-actin intensity increased from an original intensity of 14.36 ± 2.61 to 24.20 ± 3.08 in the same region (*n* = 9, *P* < 0.01, Student’s *t*-test). At 5 min, the intensity was 18.40 ± 3.00, which was still higher than that in cells without LIPUS stimulation (*n* = 9, *P* < 0.05, Student’s *t*-test). The mean fluorescence intensity of perinuclear F-actin returned to its initial level at 30 min (*n* = 9, *P* > 0.05, Student’s *t*-test) (Fig. 5c, left). In the shRNA-Piezo1 cell group, the mean fluorescence intensity of perinuclear F-actin was not significantly different at any of the time points with or without LIPUS stimulation (*n* = 9, *P* > 0.05, Student’s *t*-test) (Fig. 5c, right). Thus, these results suggest that LIPUS stimulation induces the polymerization of perinuclear F-actin in MC3T3-E1 cells and that Piezo1 is essential for this phenomenon.

## Discussion

Piezo1 expression was recently discovered at the plasma membrane in human dental pulp-derived mesenchymal stem cells,^24^ stem cells extracted from human exfoliated deciduous teeth,^25^ mouse urothelium cells,^26^ human umbilical vein endothelial cells,^19^ and HEK293T cells.^13^ Ge et al.^15^ explored the structure of the full-length mouse Piezo1 protein (2 547 amino acids) using cryoelectron microscopy with a resolution of 4.8 and found a trimeric propeller-like structure with extracellular domains resembling three distal blades and a central cap. In addition, Piezo1 is expressed in many cell types in which it conducts a variety of biomechanical stimulations and triggers different functional outcomes.^27^ Studies have shown that Piezo1 in human periodontal ligament cells plays a transduction role in the processes of mechanical stress-induced osteoclastogenesis.^28^ With mechanical force, Piezo1-dependent Ca^2+^ influx can regulate red blood cell volume.^29^ Piezo1 in mouse bladder urothelial cells can act as a mechanosensor to sense bladder fullness.^30^ Therefore, we first investigated whether Piezo1 was expressed on MC3T3-E1 cells, and clear expression of the ~260-kD Piezo1 protein was observed by western blot. The immunofluorescence results showed that Piezo1 was expressed on MC3T3-E1 cells and localized to the plasma membrane and nucleus. Both of these results indicate that Piezo1 exists in MC3T3-E1 cells.

Some studies have found that ultrasound stimulation can enhance the proliferation of preosteoblast cell lines.^31,32^ Our previous studies also demonstrated that LIPUS can facilitate cellular ingrowth into porous SiC scaffolds.^33,34^ For LIPUS to have a biological effect, the mechanical wave must be received and converted to biochemical signals, and we presume that Piezo1 is the mechanosensor in this process. To test our assumption, we next measured the proliferation and migration of MC3T3-E1 and shRNA-Piezo1 cells by wound healing and migration assays. At different time points after LIPUS stimulation, the migration and proliferation of MC3T3-E1 cells showed significant increasing tendencies compared with those of shRNA-Piezo1 cells. Therefore, we conclude that Piezo1 is a key mechanosensor in the mechanism by which ultrasound stimulation increases MC3T3-E1 cell migration and proliferation.

The trimeric propeller-shaped architecture of mouse Piezo1, as determined by electron cryomicroscopy,^15,16^ shows that Piezo1 is not only a sensor of mechanical stimuli but also a mechanosensitive cation (Ca^2+^) channel. Our previous study indicated that the intracellular calcium concentration in MC3T3-E1 cells exhibited a transient increasing trend due to the prompt response to LIPUS stimulation.^35^ Therefore, we sought to confirm that Piezo1 can be activated by LIPUS stimulation and can open for intracellular calcium. In the MC3T3-E1 cell group, the calcium fluorescence intensity traces exhibited several high peaks compared with the baseline during the period of LIPUS stimulation. The calcium influx peaks of the shRNA-Piezo1 cells were gradual, similar to those of GsMTx4-treated cells. Just as all the cationic MSCs were inhibited by GsMTx4, the cells lost the main entrance channel for Ca^2+^ when Piezo1 protein expression was knocked down, suggesting that LIPUS stimulation causes calcium influx and that calcium influx is dependent on the Piezo1 protein.

Intracellular calcium signaling is considered one of the earliest responses in osteoblasts under mechanical stimulation and can immediately initiate several essential downstream signaling pathways. Ca^2+^ is an essential second messenger in cells, and we questioned the downstream signaling processes in MC3T3 cells after calcium influx. The top candidate is ERK1/2, which is a primary signaling pathway that transmits biomechanical or biochemical signals from a variety of extracellular agents to regulate proliferation, differentiation, and more.^36–38^ In a recent study, mechanical stretching triggered prompt epithelial cell division,^20^ showing that it rapidly activated ERK1/2 phosphorylation in a Piezo1-dependent manner. In dental pulp stem cells,^39^ ERK1/2 was also shown to actively participate in cell proliferation due to ultrasound-induced stimulation. In this study, the ratio of ERK1/2 phosphorylation was significantly increased immediately after 3 min of LIPUS stimulation in the MC3T3-E1 cell group and remained at a high level for 30 min. However, in the shRNA-Piezo1 cell group, the increase was not obvious. These results show that Piezo1 could transduce LIPUS-associated mechanical signals into intracellular calcium, and Ca^2+^ could serve as a second messenger to activate ERK1/2 phosphorylation, which regulates the proliferation of MC3T3-E1 cells.

Moreover, most biological processes associated with LIPUS stimulation are accompanied by structural remodeling at the cytoskeletal level.^35,40^ The corresponding structural remodeling can also be typically mediated mechanically by direct application of physical forces such as shear stress^41^ and local mechanical force,^42^ and the fluidization response of the cytoskeleton is reversible. In our study, Phalloidin-iFluor-labeled F-actin filaments were immediately found to accumulate in the perinuclear region after LIPUS mechanical stimulation, persistently existing for 5 min and returning to their initial level at 30 min. No similar phenomenon was observed in shRNA-Piezo1 cells. These results suggest that LIPUS stimulation induces the polymerization of perinuclear F-actin in MC3T3-E1 cells and that Piezo1 is essential for this process. However, the actin rim phenomenon was not obvious, unlike in previous studies.^42,43^ Shao et al.^42^ exerted local force on the cell periphery of NIH 3T3 fibroblasts and concluded that actin reorganization and polymerization were triggered by an intracellular Ca^2+^ burst induced by the local force application. However, they found not only transient actin accumulation at the perinuclear region but also that F-actin formed a rim near the nucleus in response to the local force application. Wales et al.^43^ indicated that calcium-mediated actin reset is involved in Ca^2+^ signaling and actin dynamics under many physiological cues in response to mechanical signals. Moreover, they also observed a transient actin rim at the nucleus in Madin–Darby canine kidney epithelial cells after exposure to a shear flow of 10–20 dyn per cm^2^. Thus, this differential result may be due to the differences in cell types and mechanical forces, and further investigations of the underlying mechanism and the relationship between perinuclear F-actin accumulation and MC3T3-E1 cell proliferation are needed.

In recent studies, mechanical stimulation was shown to alter Piezo1 expression, but most of the changes occurred after a relatively long period (from 2 h to 3 d).^44–46^ To simplify the study, we assumed that LIPUS stimulation controls only the switch of Piezo1 in MC3T3-E1 cells without changing its expression. In addition, experiments investigating calcium influx and the resulting downstream signaling processes in MC3T3 cells were conducted in only the very early stage (0–30 min) after LIPUS stimulation to avoid the effect of possible changes in Piezo1 expression due to LIPUS stimulation.

There are several limitations to this study. First, we mainly focused on investigating the expression and role of Piezo1 in MC3T3-E1 cells, which are preosteoblasts, after LIPUS stimulation. However, bone trauma healing is often accompanied by the proliferation, migration, and differentiation of osteoblasts during new bone formation. Therefore, we will clarify the molecular mechanisms of Piezo1 in osteoblast differentiation and further explore the role of Piezo1 in bone formation with LIPUS stimulation. Second, Ca^2+^ signaling serves as an essential second messenger in cells that could immediately initiate downstream pathways after mechanical stimulus. Our results indicated that Piezo1 is a key mechanosensor and Ca^2+^ ion channel of MC3TC cells in response to ultrasound stimulation. However, we still need to further investigate the mechanism involved in the mechanotransduction pathway. Mitogen-activated protein kinases (MAPKs) are important transmitters of signals from the cell surface to the nucleus that jointly regulate cell growth, differentiation, stress adaptation to the environment, and other important cellular biological processes. Inhibiting Ca^2+^ signaling or MAPK will be useful for exploring the mechanism underlying the role of Piezo1 in the promotion of bone trauma repair induced by ultrasound stimulation. Moreover, we utilized only normal MC3TC cells as the control, and Piezo1 negative-control shRNA should be used as the control group to minimize the effect of lentivirus infection on the biology of MC3T3-E1 cells in future studies.

In conclusion, these results highlight the important role of Piezo1 in ultrasound-stimulated MC3T3-E1 cells. We demonstrated that Piezo1 could transduce LIPUS-associated mechanical signals into intracellular Ca^2+^ and that Ca^2+^ acted as a second messenger to activate ERK1/2 phosphorylation and perinuclear F-actin polymerization, which regulate the proliferation of MC3T3-E1 cells. This research opens new avenues into understanding how cells convert the mechanical waves of LIPUS into biochemical signals to activate biochemical pathways. The results also identify Piezo1 as a potential novel therapeutic target for fracture healing.

## Materials and methods

### MC3T3-E1 osteoblastic cell cultures

MC3T3-E1 osteoblast precursor cells (Clone 9, ATCC, Manassas, VA) were cultured in alpha-modified minimum essential medium eagle (α-MEM, Gibco, New York, NY) containing 10% (v/v) fetal bovine serum (Gibco, New York, NY) and 1% (v/v) penicillin/streptomycin (Gibco, New York, NY) in humidified incubators at 37 °C and 5% CO_2_. The complete medium was typically replaced every 2 days, and cells were subcultured via trypsinization once reaching confluency of ~90%.

### Piezo1 shRNA lentiviral particle transfection

MC3T3-E1 cells were plated in a 12-well plate at 5 × 10^4^ cells per well 24 h prior to viral infection. After the cells reached ~50% confluence, the culture medium was removed and replaced with a mixture of 1 mL of complete medium with polybrene (Santa Cruz, Dallas, Texas) at a final concentration of 5 μg·mL^−1^ and 15 μL of Piezo1 shRNA lentiviral particles (Santa Cruz, Dallas, Texas) per well. The cells were then incubated at 37 °C for another 24 h to allow viral infection and cultured in 1 mL of complete medium (without polybrene or shRNA) per well for another 24 h, allowing cell recovery and gene expression. After lentiviral particle transfection, 1 mL of α-MEM containing 1.5 μg·mL^−1^ puromycin dihydrochloride (Santa Cruz, Dallas, Texas) was added to each well to select stable clones expressing Piezo1 shRNA (shRNA-Piezo1 cells).

### Piezo1 immunofluorescence staining and imaging

MC3T3-E1 and shRNA-Piezo1 MC3T3-E1 cells were seeded into MatTek glass-bottom microwell dishes (35-mm Petri dish, MatTek, Ashland, MA) at 1 × 10^5^ cells per dish and maintained in humidified incubators at 37 °C and 5% CO_2_. The next day, the cells were rinsed with PBS twice, fixed with 4% paraformaldehyde (Lifeline Cell Technology, Frederick, MD, USA) for 8 min, and then permeabilized with 0.1% Triton X-100 (MP Biomedicals, Solon, OH, USA) for 10 min at room temperature. After this, the cells were blocked with 5% goat serum (Thermo Fisher Scientific, Waltham, MA, USA) in PBS to prevent nonspecific binding for 1 h at room temperature. Then, they were incubated with a primary antibody against Piezo1 (1:100, Novus Biologicals, Littleton, CO, USA) at 4 °C overnight. After washing three times with PBS containing 0.1% Triton X-100, the cells were incubated with a goat anti-rabbit IgG H&L (Alexa Fluor 488) (1:1 000, Abcam, Cambridge, MA, USA) secondary antibody for 1 h at room temperature. The cells were washed as described above and then incubated with DAPI nuclear stain (Thermo Fisher Scientific, Waltham, MA, USA) for 10 min at room temperature. Following staining, the cells were imaged on a Zeiss Axiovert 200 M (LSM 510 META) laser scanning confocal microscope (Carl Zeiss, Germany).

### Low-intensity ultrasound stimulation

**Fig. 6:**
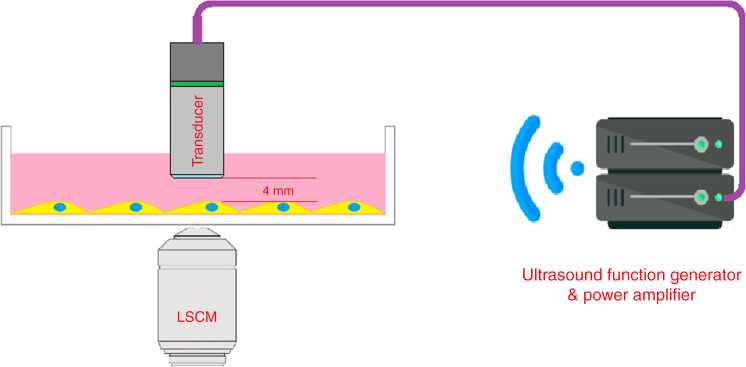
Schematic diagram of the experimental setup for the LIPUS stimulation of MC3T3-E1 cells. The setup includes a function generator, a radio-frequency power amplifier, a transducer, and a laser scanning confocal microscope (LSCM). The surface of the transducer was immersed in the medium and located 4 mm away from the cells

The LIPUS signal was administered by a function generator (AFG3021, Tektronix Inc, Beaverton, OR) with a 1-Hz pulse repetition frequency, 20% duty cycle, 200-mV amplitude, and 2.25-MHz burst sin wave and amplified by a radio-frequency power amplifier (E&I 2100 L, Electronics & Innovation, Ltd., Rochester, NY) to drive the 2.25-MHz transducer activation (Shinjuku, Tokyo, Japan). The transducer element was 6 mm in length. During the experiment, the surface of the transducer was immersed in the medium and located 4 mm away from the cells, which were attached to the bottom of the dishes and plates (Fig. 6). The beam pattern of the transducer was quantified before the studies. The 2.25-MHz transducer was stabilized on a customized three-dimensional stage, and the acoustic energy map was determined by a “Golden Lipstick” hydrophone (HGL-0400, ONDA Corp., Sunnyvale, CA). The acoustic energy transmitted to the cells was ~40 mW. The total time of the calcium oscillation experiment was 6 min, including 1 min of baseline stimulation, 3 min of active stimulation, and 2 min of regression. In all the other experiments herein, the LIPUS stimulation time was 3 min.

### Wound healing and migration assay

To measure the migration and proliferation of MC3T3-E1 and shRNA-Piezo1 cells with LIPUS stimulation, a wound healing and migration assay was conducted, with the characteristic parameter being the change in the cell-covered area over time. Each group of cells was seeded onto a dish (Culture-Insert 2 Well in μ-Dish, 35 mmm ibidi, Martinsried, Germany) at a density of 1 × 10^4^ cells per well and then incubated at 37 °C and 5% CO_2_ for 24 h. Once the cells reached 100% confluence, the Culture-Insert, which provided a 500-μm-thick wall that separated the cultured cells, was removed. The LIPUS stimulation time was 3 min, and the LIPUS transducer was also placed in the control group dishes without power. Random images of the gap at 0, 4, 8, 12, and 24 h after LIPUS stimulation were obtained using a Zeiss Axio Observer D1 phase-contrast microscope (Carl Zeiss, Germany). All these experiments were independently performed three times. The changes in the gap area were measured and analyzed using ImageJ software (National Institutes of Health, Bethesda, MD, USA).

### Fluorescence imaging of calcium oscillation

Three groups of cells were evaluated in this experiment: MC3T3-E1 cells, shRNA-Piezo1 cells, and MC3T3-E1 cells treated with the nonselective cationic MSC inhibitor GsMTx4 (GsMTx4-treated cells). To evaluate the changes in intracellular calcium concentrations, a calcium-sensitive fluorescence indicator, CalciFluor™ Fluo-8, AM (Ex = 490 nm, Em = 520 nm, Santa Cruz, Dallas, Texas), was used to stain the cells according to the manufacturer’s instructions. Briefly, confluent 35-mm MatTek glass-bottom microwell dishes containing cells were first washed twice in DPBS and then stained with 2 μmol·L^−1^ CalciFluor™ Fluo-8 AM in DPBS at 37 °C and 5% CO_2_ for 10 min. The cell dishes were shielded from light in aluminum foil. Before ultrasound stimulation, 1.5 mL of α-MEM was added to cells in the MC3T3-E1 and shRNA-Piezo1 groups. In the GsMTx4 inhibition experiments, 1.5 mL of α-MEM with GsMTx4 (TOCRIS, MN, USA) at a concentration of 4 μmol·L^−1^ was added to the dishes. Next, the cells loaded with CalciFluor™ Fluo-8 AM (Fluo-8) were visualized and imaged to observe calcium oscillations using a Zeiss Axiovert 200 M (LSM 510 META) laser scanning confocal microscope. The total time of the calcium oscillation experiment was 6 min, including 1 min at baseline without LIPUS stimulation, 3 min of active stimulation, and 2 min of regression. Time-lapse sequences were collected every 1.8 s for 6 min. We found that not all the cells were sensitive to LIPUS stimulation. This phenomenon also appeared in the study by Taifeng et al.^47^ (fluid shear stress stimulation of mouse bone marrow stromal cells) and may be related to the location and status of the cells. Therefore, we chose ten active Ca^2+^ oscillating cells within each field of view for the image analysis. In all the groups in this experiment, the fluorescence intensities of ten cells in each field were quantified using LSM Image Browser software (version 4.2, Carl Zeiss, Germany). The fluorescence intensities of these cells reflected the characteristics of each group in regards to the calcium influx caused by LIPUS stimulation.

### Western blot analysis

MC3T3-E1 and shRNA-Piezo1 cells were seeded in 6-well culture plates at 1 × 10^5^ cells per well and incubated in modified complete α-MEM in a humidified atmosphere of 5% CO_2_ and 37 °C. The next day, the cells were divided into the control group and LIPUS treatment group. LIPUS stimulation was generated by a Sonicator^®^740 instrument (Mettler Electronics, CA, USA) in the study. The 1-MHz US transducer (ME7410) was triggered by an ultrasound signal comprising a sinusoidal ultrasound pulse frequency of 1 MHz, a duty cycle of 20%, and a repetition rate of 100 Hz. The spatial average-temporal average acoustic energy delivered to the cells was ~30 mW.^33^ An acoustic gel was also applied between the transducer and culture plates to facilitate the transmission of acoustic energy. For the LIPUS treatment group, the Sonicator^®^740 was turned on for 3 min of stimulation. However, it was turned off for the control group.

At various time points (0, 5, and 30 min) following LIPUS treatment, the cells were washed twice with ice-cold Tris-buffered saline (TBS, Bio-Rad, Hercules, CA, USA), lysed in RIPA buffer (Cell Signaling Technology, Danvers, MA, USA), and analyzed using the Bradford assay. Twenty micrograms of protein mixed with an equal volume of 2x Laemmli Sample Buffer (Bio-Rad, Hercules, CA, USA) was electrophoresed on 4%–20% precast polyacrylamide gels (Bio-Rad, Hercules, CA, USA). The proteins were then transferred from the gel to a polyvinylidene difluoride (Bio-Rad, Hercules, CA, USA) membrane at 100 V for 70 min. After blocking with Odyssey Blocking Buffer (LI-COR Biosciences, Lincoln, NE, USA), the membrane was incubated overnight at 4 °C with rabbit polyclonal antibodies targeting GAPDH (1:5 000, Cell Signaling Technology, Danvers, MA, USA), p44/42 MAPK (ERK1/2) (1:2 000, Cell Signaling Technology, Danvers, MA, USA), and phospho-p44/42 MAPK (p-ERK1/2) (1:2 000, Cell Signaling Technology, Danvers, MA, USA). Then, the membrane was thoroughly rinsed with TBS Tween-20 (TBST containing 0.1% Tween-20) three times and incubated with goat anti-rabbit IgG conjugated with a StarBright Blue 520 fluorophore (Bio-Rad, Hercules, CA, USA) at room temperature for another 1 h. Visualization was performed using the LI-COR Odyssey CLx scanner and software (LI-COR Biosciences, Lincoln, NE, USA). Rabbit polyclonal antibodies targeting Piezo1 (1:2 500, Novus Biologicals, Littleton, CO, USA) were used for the detection of Piezo1 in MC3T3-E1 and shRNA-Piezo1 cells by western blot, and the same protocol was used without ultrasound stimulation application. Proteins were quantified using ImageJ software. The expression of Piezo1 is presented as the normalized ratio of the target protein to GAPDH (Piezo1/GAPDH). Based on both ERK1 and ERK2, the relative expression of the protein doublet (p-ERK1/2 vs. ERK1/2) is presented as a parameter of ERK1/2 activation. All experiments were performed independently three times.

### Perinuclear F-actin staining

MC3T3-E1 and shRNA-Piezo1 MC3T3-E1 cells were seeded into MatTek glass-bottom microwell dishes at 1 × 10^5^ cells per dish and cultured in humidified incubators at 37 °C and 5% CO_2_ for 24 h. After the cells were stimulated by LIPUS for 3 min, they were promptly fixed with 4% paraformaldehyde at the following time points: 0, 5, and 30 min. The cells were permeabilized with 0.1% Triton X-100 for 10 min at room temperature and then rinsed with DPBS three times. Following fixation and permeabilization, the cells were blocked with 5% goat serum in PBS and stained with Phalloidin-iFluor 555 Reagent (1:1 000, Abcam, Cambridge, MA, USA), which can bind to F-actin, for 20 min at room temperature. After a quick wash with DPBS, the cells were incubated with DAPI nuclear stain for 10 min at room temperature. Finally, the cellular F-actin filaments were imaged with a Zeiss Axiovert 200 M (LSM 510 META) confocal microscope (Carl Zeiss, Germany). The mean fluorescence light intensity of F-actin was measured and analyzed within the perinuclear regions (1-μm range around the nucleus) using ImageJ software. All experiments were conducted three times independently, and a total of nine cells from each group at specific time points were selected for F-actin intensity measurement.

### Statistical analysis

The results in the study are expressed as the mean ± standard deviation. The differences in the abovementioned measurements between groups were compared using Student’s *t*-test. All statistical analyses were performed with SPSS version 13 software (SPSS Inc., Chicago, IL). Statistical significance was set at *P < 0.05 and **P < 0.01.

## Supplementary information

Supplementary figure1
