# Supplementary figures and images for: Piezo1 channel activation in response to mechanobiological acoustic radiation force in osteoblastic cells
Source: Bone Res. 2021 Mar 10;9:16. doi: 10.1038/s41413-020-00124-y (PMC7946898; doi:10.1038/s41413-020-00124-y)

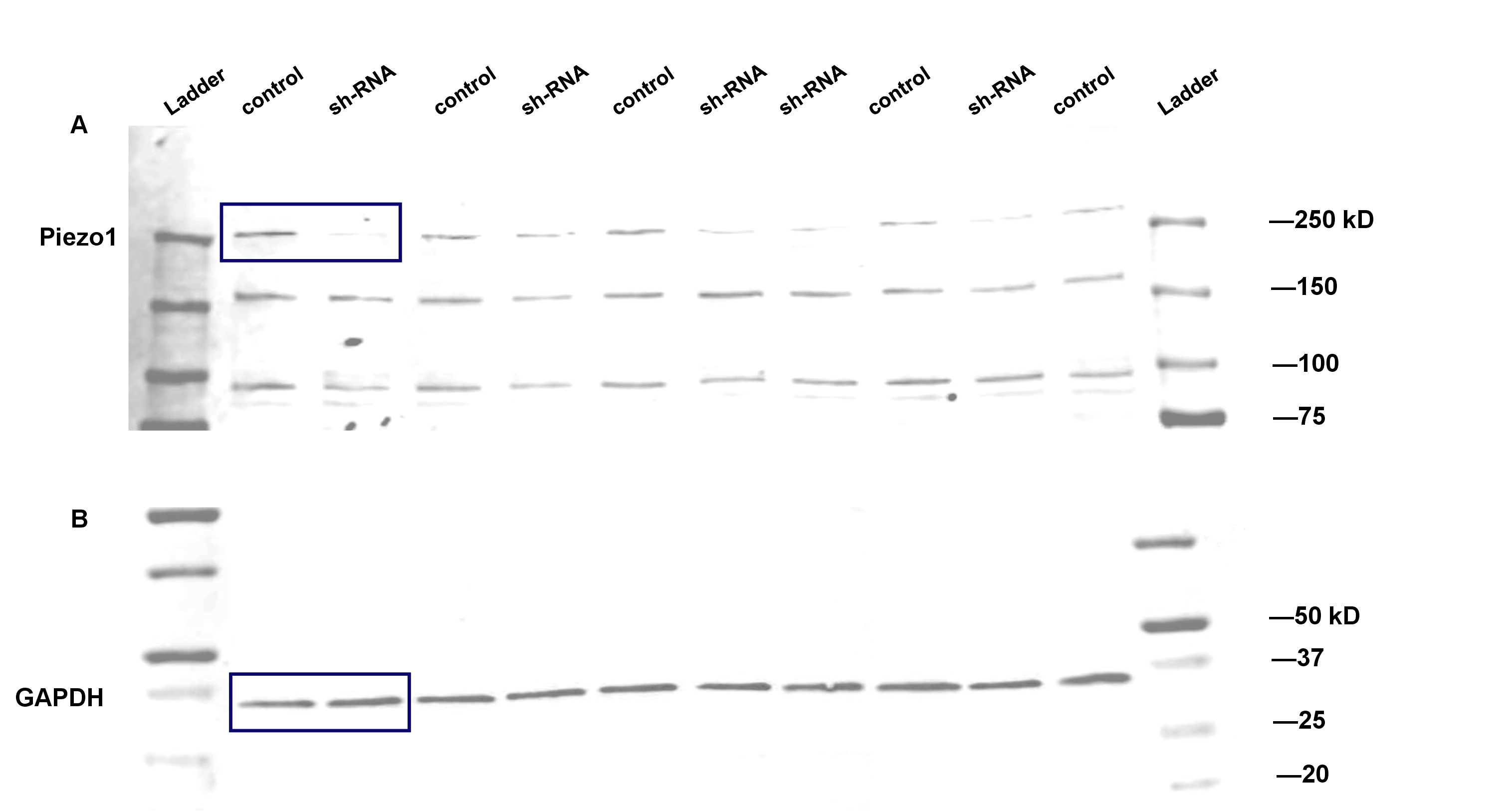

Supplement: Supplementary file 1 — Supplementary figure1 [file 41413_2020_124_MOESM1_ESM.tif]
